# Primary carcinoma of the lung in von Recklinghausen neurofibromatosis

**DOI:** 10.4103/0970-2113.56348

**Published:** 2009

**Authors:** K. B. Gupta, Vipul Kumar, Sanjeev Tandon, Meenu Gill

**Affiliations:** *Department of TB and Respiratory Medicine, Pt .B. D. Sharma University of Health Sciences, Rohtak, Haryana, India*; 1 *Department of Pathology, Pt .B. D. Sharma University of Health Sciences, Rohtak, Haryana, India*

**Keywords:** Neurofibromatosis, primary carcinoma of lung, von Recklinghausen's disease

## Abstract

von Recklinghausen neurofibromatosis (NF-1) is the most common inherited syndrome predisposing to neoplasia, particularly neural crest-derived tumors. However, lung malignancies reported in association with neurofibromatosis are sparse. We present a case of a 48-year-old man with NF-1 that manifested as carcinoma of lung, in order to discuss the linkage between these two entities.

## INTRODUCTION

von Recklinghausen neurofibromatosis is a member of phakomatoses or neurocutaneous syndromes, all of which have both neurologic and dermatologic lesions. NF-1 is an autosomal dominant disease caused by mutation of a gene on the long arm of chromosome 17, which encodes a protein known as neurofibromin, a negative regulator of Ras oncogene. The classical or peripheral form of the disease, also known as neurofibromatosis type-1 (NF-I), accounts for 85% of all cases. Its cardinal features are café-au-lait macules and multiple neurofibromas. The presence of six or more café-au-lait macules over 5 mm in prepubertal individuals and over 15 mm in post-pubertal individuals is diagnostic of NF-1. Other features include two or more Lisch nodules (Iris hamartomas), axillary or inguinal freckling, sphenoid dysplasia, and pseudoarthrosis of tibia.[1] Patients with NF-1 are at increased risk of developing nervous system neoplasms.[2] Even though it is known to be the most common inherited syndrome in man predisposing to neoplasia, occurrence of malignant tumors other than neurogenic tumors is not common.[2–5]

## CASE REPORT

**Figure 1:**
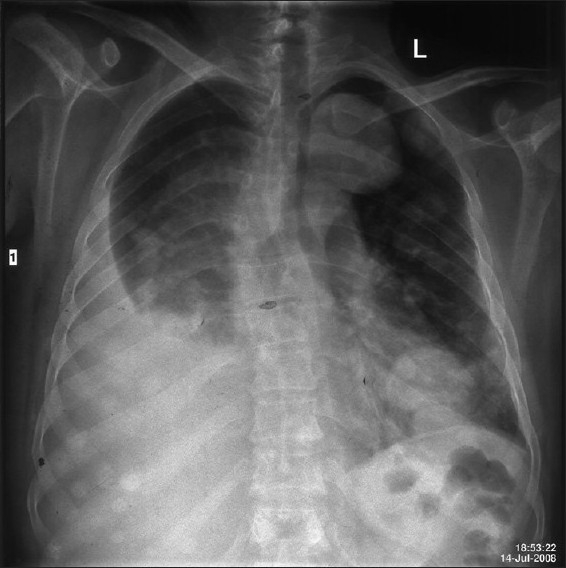
Chest X-ray showing nodular opacities in bilateral lung fields and pleural effusion on right side

**Figure 2:**
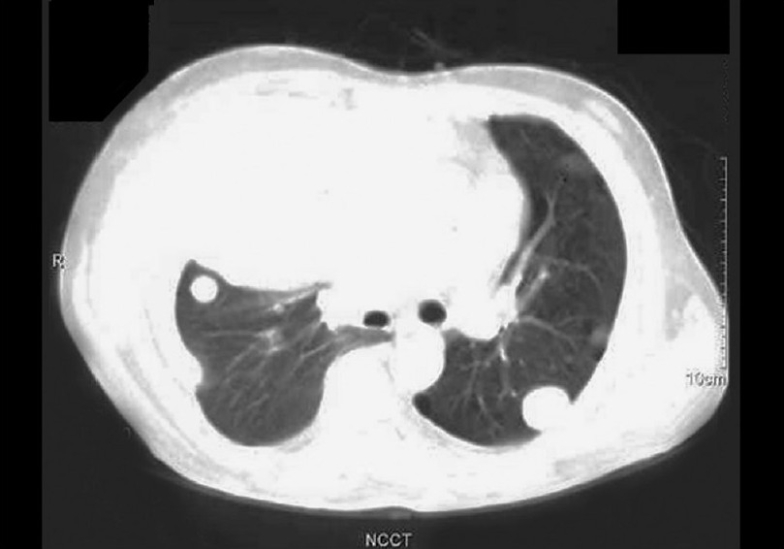
CT scan showing mass lesion in right upper zone with metastasis in both lung fields

**Figure 3:**
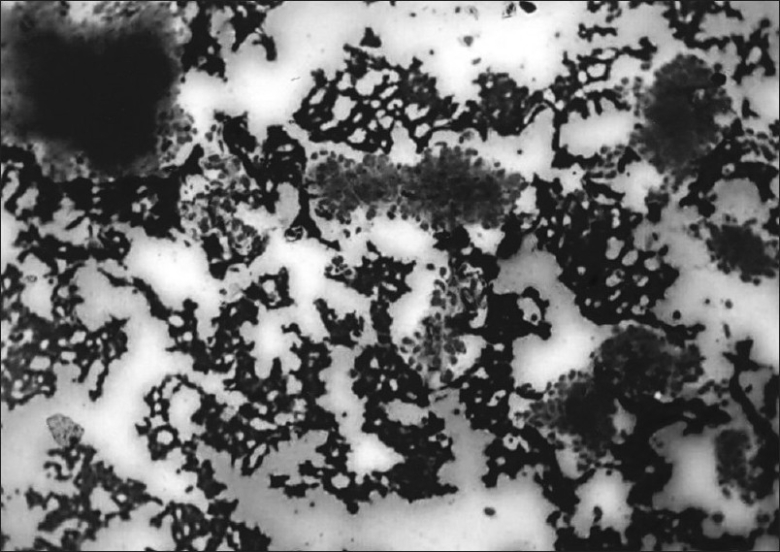
Photomicrograph showing groups of malignant cells (100×)

A 48-year-old man was admitted with history of fever since 45 days, cough with sputum since 10 days, and shortness of breath since 2 days. There were no remarkable data in his medical and family histories. He was a chronic smoker and alcoholic with mixed food habits. He was a photographer by occupation and did not have any environmental or occupational asbestos exposure. Physical examination revealed a number of café-au-lait spots and multiple cutaneous neurofibromas, suggestive of NF-1. His chest X-ray showed nodular opacities in bilateral lung fields and pleural effusion on the right side [Figure 1]. Contrast enhanced CT scan of chest revealed a heterogeneously enhancing mass lesion in the right upper zone with volume loss, multiple heterogeneously enhancing rounded opacities of variable size in both lung fields, and pleural effusion on the right side. No mediastinal lymphadenopathy was seen [Figure 2]. CT scan of head revealed a focal hypodensity in genu of internal capsule, suggestive of Lacunar infarct. Cytological examination of pleural fluid revealed atypical cells showing marked degenerative changes in overlapping clusters and forming papillae. These cells had high nuclear-to-cytoplasmic ratio and were highly suspicious of malignancy. Pleural biopsy done twice by Coopes' needle revealed only chronic inflammation. Biochemical features of pleural fluid were protein- 5.4 g/ dL, LDH-1518 U/L (normal range, 240-480 IU/L), and amylase-28 U/L. FNAC of lung mass was performed, which revealed poorly differentiated carcinoma of lung [Figure 3].

## DISCUSSION

The most common pulmonary manifestations of neuro fibromatosis consist of diffuse interstitial fibrosis and bullae formation with a prevalence rate of about 10% and 20%, respectively.[1,6]

It has been suggested that the genetic abnormality responsible for NF-1 increases a patient's risk of various kinds of malignancies, and therefore individuals with NF-1 have a significantly higher incidence of malignant schwannoma, neurofibrosarcoma, intracranial glioma, and pheochromocytoma.[7] However, association of primary lung carcinoma with NF-1 is not common.[5] A Japanese review reported only 11 cases of NF-1 with primary lung carcinoma until 1992.[5] Poorly differentiated tumor was observed in seven out of eight patients.[5] We found a few more cases in the medical literature presenting this association.[3,4,8–10] The question from these reports is whether the association is coincidental or there is an increased risk of malignancy in NF-1 patients. Since NF-1 is the most common inherited syndrome predisposing to neoplasia and individuals with NF-1 have significantly higher incidence of neural crest–derived tumors, there might be also an increased risk for the development of primary lung carcinoma in NF-1 patients.[7] NF-1 gene has been mapped to a small region of chromosome 17 q.[2] Therefore, many surveys were conducted to show any genetic linkage between the abnormalities on chromosome 17 and malignant transformation in NF-1.[3,4,11] In a study by Menon *et al.,*[11] deletions of chromosome 17 in NF-1-derived tumor specimens were searched. Researchers found 17p deletions in neurofibrosarcomas. This region is known to contain *p53* gene, which has been implicated in progression of a broad spectrum of human cancers, including lung carcinomas.[3] In a study by Shimizu *et al.,*[3] loss of heterozygosity in chromosome arm 17 p but not 17 q was observed in a patient with small cell lung carcinoma with NF-1. They suggested that inactivation of tumor suppressor gene on chromosome 17 p, most likely *p 53*, might be responsible for development of small cell lung carcinoma in their patient.

On the basis of these findings, one can speculate that the risk for the development of primary lung carcinoma in NF-1 patients might be increased, at least in some patients, due to increased prevalence of deletions on chromosome arm 17 p.

## CONCLUSION

The present case illustrates the intricacy of management of patients with NF-1 and pulmonary involvement. As routine screening for gliomas is a part of care of patients with NF-1, it is suggested that screening for lung cancer in this population should be a routine.
